# A practical guide to the management of dyslipidaemia

**DOI:** 10.1007/s00392-025-02833-y

**Published:** 2026-01-08

**Authors:** Patrick M. Siegel, Julius L. Katzmann, Julia Weinmann-Menke, Ulf Landmesser, Heribert Schunkert, Stephan Baldus, Michael Böhm, Ulrich Laufs, Thomas F. Lüscher, Ingo Hilgendorf

**Affiliations:** 1 Department of Cardiology and Angiology, University Heart Center Freiburg-Bad Krozingen, Freiburg, Germany; 2 Faculty of Medicine, University of Freiburg, Freiburg, Germany; 3 Department of Cardiology, University Hospital Leipzig, Leipzig, Germany; 4 Department of Internal Medicine I and Research Center of Immunotherapy, University Medical Center Mainz, Mainz, Germany; 5 Department of Cardiology, Angiology and Intensive Care Medicine, Deutsches Herzzentrum der Charité; Charité-Universitätsmedizin Berlin, Berlin, Germany; 6 German Center for Cardiovascular Research (DZHK), Partner Site Berlin, Berlin, Germany; 7 Friede Springer Cardiovascular Prevention Center@Charité Berlin, Berlin, Germany; 8 Department of Cardiology, Deutsches Herzzentrum München, Universitätsklinikum der Technischen Universität München, Munich, Germany; 9 DZHK (German Centre for Cardiovascular Research), Partner site Munich, Munich Heart Alliance, Munich, Germany; 10 Department of Cardiology, Heart Center, University Hospital Cologne, Cologne, Germany; 11 HOMICAREM (HOMburg Institute for CArdioREnalMetabolic Medicine), Homburg/Saar, Germany; 12 Department of Internal Medicine III, Cardiology, Angiology, Intensive Care Medicine, Universitätsklinikum des Saarlandes, Saarland University, Homburg/Saar, Germany; 13 Heart Division, Royal Brompton and Harefield Hospital GSTT and King’s College, London, UK; 14 Center for Molecular Cardiology, University of Zurich, Schlieren, Switzerland

**Keywords:** Lipoprotein(a), LDL cholesterol, Triglycerides, Cardiovascular risk, Lipid-lowering drugs

## Abstract

Dyslipidaemia, especially elevated low-density lipoprotein cholesterol (LDL-C), is a major modifiable risk factor for atherosclerotic cardiovascular disease (ASCVD). Dyslipidaemia remains underdiagnosed and undertreated. Dyslipidemia is highly prevalent in Germany. Even among patients with high- and very-high cardiovascular risk, LDL-C targets are often not achieved. This paper highlights key lipid parameters beyond LDL-C, such as triglycerides and lipoprotein(a), which contribute to residual cardiovascular risk. Practical guidance to address diagnostic challenges and cardiovascular risk assessment, especially in younger adults and those with risk modifiers, is provided. Lifestyle interventions are the basis of therapy. Statins remain the first-line treatment, with additional options including ezetimibe, bempedoic acid, and PCSK9 inhibitors, alone or in combination. Novel lipid-lowering therapies are currently in development and may offer more individualized treatment options in the future. The most important messages from the 2025 Focused Update of the 2019 ESC/EAS guidelines for the management of dyslipidaemias have been incorporated into the paper. While LDL-C targets remain unchanged, important novel recommendations encompass consideration of cardiovascular risk modifiers such as lipoprotein(a) and CRP/inflammatory diseases. A second important new recommendation is the use of potent early combination therapy after an acute coronary syndrome. Improved awareness, early diagnosis, and evidence-based lipid management are critical for reducing ASCVD burden. This paper is aimed at supporting clinicians in optimizing lipid diagnostics and therapy in daily practice.

**Graphical Abstract:**

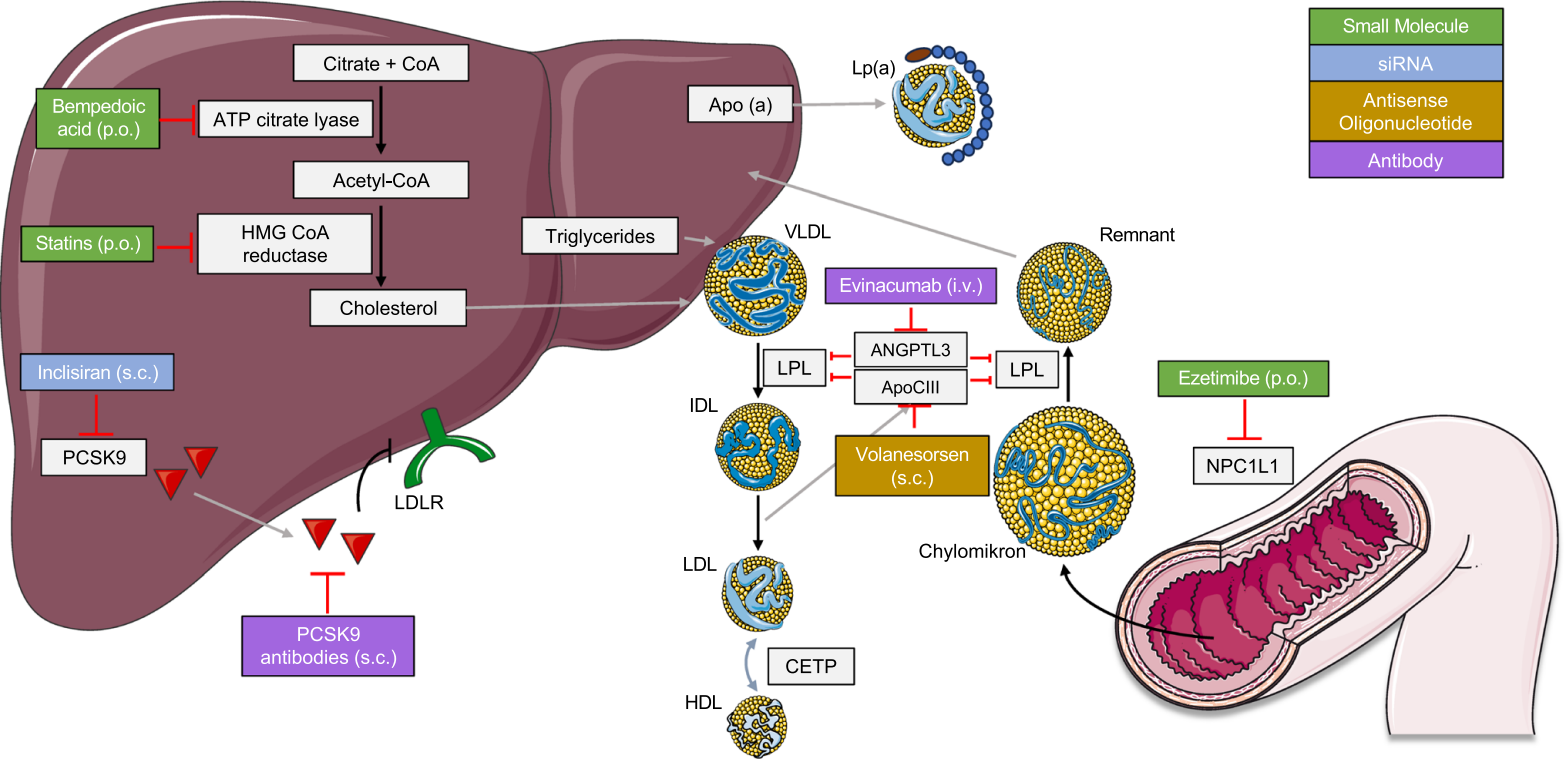

## Epidemiology and cardiovascular risk associated with lipids

Atherosclerotic cardiovascular disease (ASCVD) remains the leading cause of death worldwide, and dyslipidaemia, including hypercholesterolaemia, stands out as a causal risk factor for the development of atherosclerosis. The prevalence of hypercholesterolaemia in Europe is high, with significant variability between countries and populations depending on the specific criteria and populations studied. According to the German Health Interview and Examination Survey for Adults (DEGS1), the overall prevalence of dyslipidaemia in Germany (with total cholesterol ≥ 190 mg/dL and 240 mg/dL being considered “elevated” and “highly elevated,” respectively) is 64.5% for men and 65.7% for women, with more than half of these cases being previously undiagnosed [1–3]. But there is also an increasing burden of hypercholesterolaemia and ASCVD in Iow- and middle-income countries [4].

Despite the central role of low-density lipoprotein cholesterol (LDL-C) for the development of ASCVD and the availability of effective lipid-lowering drugs, healthcare providers are currently not addressing this condition adequately. For instance, in the primary prevention setting, the PROCYON survey revealed that a significant proportion of patients did not receive lipid-lowering medication, and many did not achieve LDL-C targets even when treated [5]. Even more concerning, in secondary prevention, statin utilization was low and only 30% of cardiovascular high- and very high-risk patients achieved guideline-recommended LDL-C targets [6]. This problem is ongoing, as shown in the more recent “Lipid-Snapshot-Study” based on data from 2022–2023 in Germany. Only 27% of patients with ASCVD treated by office-based cardiologists achieved the guideline-recommended target of < 55 mg/dl for low-density lipoprotein cholesterol (LDL-C). This figure was worse for ASCVD patients managed by general practitioners (GPs), who only achieved their LDL-C targets in around 12%. Around 26% of patients managed by GPs vs. only 1.5% of those managed by cardiologists received no lipid-lowering therapy. These data highlight the need for increased awareness regarding the risks and the management of dyslipidaemia in Germany [7].

Here, we will present the clinically most important subsets of the different lipoproteins and lipids measurable in blood, which stand out as relevant for cardiovascular risk assessment and management.

Apolipoprotein B (ApoB): *Apolipoprotein B (ApoB)* is the main structural protein of all atherogenic lipoproteins, including LDL, very-LDL (VLDL), and lipoprotein(a) [Lp(a)]. ApoB provides a direct measure of the total number of atherogenic lipoprotein particles—including LDL, very-low-density lipoproteins (VLDL), intermediate-density lipoproteins (IDL), and lipoprotein(a)—since each of these particles contains exactly one ApoB molecule. In contrast, LDL-C quantifies the cholesterol content within LDL particles, which can vary widely between individuals and particle sizes. Therefore, in conditions with a conflict between LDL-C and particle number—such as metabolic syndrome, diabetes, obesity, or hypertriglyceridemia—apoB more accurately reflects the true atherogenic burden. Moreover, ApoB has been shown in multiple epidemiologic and Mendelian randomization studies to be a stronger predictor of cardiovascular events than LDL-C, especially when discordance exists among lipid markers. ApoB measurement is also analytically robust, since it is not affected by fasting status or triglyceride levels, and does not require complex estimation formulas. However, since most trials used LDL-C as a therapeutic target, it is still considered the diagnostic standard [8–10].

Low-density lipoprotein cholesterol: In most instances, the majority of ApoB-containing lipoproteins are *low-density lipoprotein* (LDL) particles (up to 90% during normal conditions [11]). LDL is the major target for prevention of ASCVD. Elevated LDL-C levels are causally linked to atherosclerosis and cardiovascular events. Moreover, there is a log-linear relation between the absolute plasma LDL-C changes and the risk of cardiovascular disease [12, 13]. Additionally, in established ASCVD, circulating LDL-C levels are proportional to the rate of atherosclerotic plaque progression [14, 15]. However, even in the presence of non-elevated LDL-C levels, atherosclerotic lesions can form given the widespread genetic disposition for these diseases [16, 17].

Remnant cholesterol: *Remnant cholesterol* refers to the cholesterol content within triglyceride-rich lipoproteins (TRLs), including VLDL, intermediate-density lipoproteins (IDL), and chylomicron remnants. Several definitions of remnant cholesterol have been proposed [18]. Remnant cholesterol is an independent risk factor for ASCVD, contributing to residual cardiovascular risk even when LDL-C levels are well controlled [19–21].

Triglycerides (TG): *Triglycerides (TG)* are primarily carried by VLDL particles, which are synthesized in the liver. Elevated triglyceride levels are associated with increased ASCVD risk; however, interventional studies have not demonstrated a clear risk reduction of ASCVD by lowering TG levels. VLDL particles undergo lipolysis to form IDL and subsequently LDL. The available evidence suggests that the causal effect of TRLs and their remnants on the risk of ASCVD is determined by the circulating concentration of ApoB-containing particles, rather than by the TG content itself. Hypertriglyceridaemia is diagnosed if fasting TG levels are exceeding 150 mg/dL, while levels can fluctuate depending on lifestyle and comorbidities [12, 22, 23]. TG levels > 500 mg/dL gradually raise the risk of pancreatitis [24].

*HDL cholesterol (HDL-C)*: Low HDL-C serum concentrations are an indicator of metabolic and inflammatory diseases such as diabetes, metabolic syndrome, or smoking and correlate with a higher risk of ASCVD. Lifestyle changes such as physical exercise that increase HDL-C are beneficial; however, pharmacotherapies that increase HDL-C serum concentrations without lowering ApoB have not shown ASCVD risk reduction [25, 26].

*Lipoprotein(a) [Lp(a)]:* Lipoprotein(a) is an LDL-like particle with an additional protein, apolipoprotein(a), covalently bound to its ApoB component. Elevated Lp(a) levels are primarily genetically determined. It has been proposed that Lp(a) exerts pro-atherogenic, pro-inflammatory, and pro-thrombotic effects. Observational and genetic evidence convincingly demonstrates that elevated Lp(a) contributes to the development and progression of ASCVD, aortic valve stenosis, and cardiovascular and all-cause mortality in men and women and across ethnic groups [27, 28].

## Diagnostics

Correct measurement of plasma lipids provides the basis for subsequent management. However, there are several pitfalls which can hinder accurate quantification of plasma lipids. We would therefore like to highlight several important points.

A full lipid profile should include the following parameters: total cholesterol (TC), HDL-C, LDL-C, and triglycerides. TC and HDL-C are required for risk assessment using the SCORE2 calculations (see risk assessment below).

Some laboratories calculate LDL-C by the “Friedewald-Formula”: LDL-C (mg/dL) = TC − HDL-C − (TG/5). Although this is convenient, it is prone to measurement errors as it assumes a constant cholesterol/TG ratio in VLDL. Particularly in high TG concentrations (> 400 mg/dL), the Friedewald-Formula cannot be used. Instead, direct enzymatic measurement of LDL-C is recommended [29]. LDL-C concentrations can be presented in mg/dL or mmol/L; to convert LDL-C between both units, the following formula can be applied: LDL-C (mg/dL) = LDL-C (mmol/L) × 38.67. We recommend physicians be aware of the measurement methods used by their laboratory.

In patients with high TG levels, even direct measurement of LDL-C may be inaccurate [30]. Such elevated TG levels may occur in the non-fasting state, in patients with diabetes mellitus, metabolic syndrome, and obesity. Thus, if high levels of TG (> 400 mg/dL) are measured, it is recommended to calculate non-HDL-cholesterol (non-HDL-C = TC − HDL-C) and/or measure ApoB levels directly [29]. Although TG levels may be elevated, non-fasting samples are sufficient, as non-fasting lipid profiles are more convenient in clinical practice, have better patient acceptance, and have the same prognostic value as fasting samples [31].

Lp(a) measurement should be measured at least once for every patient. Several considerations should be kept in mind when measuring Lp(a):

- Repeat measurements are only required in patients with comorbidities (e.g., infection, kidney or liver disease), women after menopause (if initial measurement was before menopause), or children with stroke, as levels may fluctuate in these groups [28].
- Ideally, Lp(a) levels should be measured using an assay that targets a unique, non-repetitive epitope of apolipoprotein(a), ensuring that each Lp(a) particle is counted only once and reported in nmol/L. However, because developing such highly specific antibodies is technically challenging, most currently available assays use polyclonal antibodies that bind to multiple epitopes. This can result in inaccuracies—underestimating Lp(a) concentrations in individuals with smaller apolipoprotein(a) isoforms and overestimating them in those with larger isoforms. As a result, polyclonal antibody-based assays are not suitable for reporting in true molar units. While calibration against isoform-insensitive reference methods may allow approximation in nmol/L, this is not always feasible, and in most cases, values should be reported in mg/dL. Due to substantial variability between assays, a universal conversion factor between mg/dL and nmol/L cannot be applied. As a rough estimate, a conversion factor between 2 and 2.5 may be used to switch from mg/dL to nmol/L, but this should be interpreted with caution [28].
- Lp(a) carries cholesterol, which is included in LDL-C measurement. Earlier studies examining isolated Lp(a) particles indicated that cholesterol comprises approximately 30% to 45% of the total Lp(a) mass. Based on this, Lp(a) cholesterol has often been estimated by multiplying the Lp(a) mass concentration (mg/dL) by 0.3. This estimate is then used to adjust LDL-C levels, yielding the Lp(a) cholesterol-corrected LDL-C value. However, Lp(a) cholesterol varies between 6 and 60% [32]. Consequently, no correction should be applied, particularly when setting LDL-C treatment targets. In certain clinical situations, a correction for Lp(a) cholesterol may be applied, such as suspected familial hypercholesterolemia, where a correction of LDL-C may avoid unnecessary sequencing, or in patients with elevated Lp(a) and presumed reduced statin responsiveness [28]. In the future, this issue may be solved by direct measurement of Lp(a) cholesterol using LPA4-magnetic beads directed to apolipoprotein(a) [33].
- A solution to harmonize the different assays that are in clinical use could be to express Lp(a) in deciles instead of absolute units.

## Assessing and managing cardiovascular risk

As indicated above, hypercholesterolaemia is a critical risk factor for ASCVD; however, total cardiovascular risk is the result of multiple, interacting risk factors. The interplay between these risk factors can be synergistic, meaning the presence of multiple risk factors exponentially increases the overall cardiovascular risk [34, 35]. For instance, individuals with both hypercholesterolaemia and hypertension have a higher incidence of coronary events than those with either condition alone [36]. Interestingly, risk factors also multiplicatively interact with genetic disposition, such that—vice versa—treatment of modifiable risk factors profoundly lowers total risk [37, 38].

Therefore, comprehensive risk assessment and management should also include other risk factors such as hypertension, diabetes, smoking, chronic kidney disease, and obesity. This concept is reflected in the recent update of the 2019 guidelines for the management of dyslipidaemias. In apparently healthy individuals, cardiovascular risk should be assessed using the SCORE2 risk score (< 70 years) or the SCORE2 for older people (SCORE2-OP; ≥ 70 years) [39]. Meanwhile, SCORE2-diabetes (SCORE2-DM) should be used in individuals with type 2 diabetes mellitus who are aged 40–79 years and do not have a history of ASCVD or severe target-organ damage [40]. These scores give an estimate of the 10-year risk of fatal and non-fatal cardiovascular events, while ≥ 20% indicates “very-high risk,” whereas levels between ≥ 10% but < 20% are considered “high risk” [39].

In patients with comorbidities such as chronic kidney disease (GFR < 60 mL/min/1.73 m^2^), diabetes mellitus (with other risk factors, > 10-year duration, or target organ damage), familial hypercholesterolaemia, or documented ASCVD, cardiovascular risk does not need to be calculated. Rather, these patients will be automatically assigned to the “high-risk” or “very-high-risk” categories [29, 36].

Depending on the risk category, LDL-C treatment targets are assigned. The LDL-C targets remained unchanged in the current guideline update compared to the 2019 guidelines. The following LDL-C targets are recommended:

- Extreme risk: < 40 mg/dL
- Very-high risk: < 55 mg/dL and at least 50% reduction from baseline
- High risk: < 70 mg/dL and at least 50% reduction from baseline
- Moderate risk: < 100 mg/dL
- Low risk: < 116 mg/dL

The 2025 EAS/ESC dyslipidaemia guideline update highlights the “extreme risk” category, which was introduced in the 2019 guideline, which includes patients with recurrent events despite lipid-lowering therapy and patients with polyvascular disease. For these patients, an LDL-C target of < 40 mg/dL is recommended [39]. This category will be applicable to many patients in daily practice. However, such low LDL-C levels may not be achievable with oral agents alone in a vast majority of patients. Furthermore, guidance for the primary prevention setting is provided [39]. A step-wise approach (as described in the 2021 ESC prevention guidelines) with an initial treatment target of LDL-C < 100 mg/dL should be reserved only for low- and intermediate-risk patients [36].

Several considerations need to be made when assessing lipid levels and their relation with cardiovascular risk.

### Patients with familial hypercholesterolaemia (FH)

Heterozygous FH is relatively common (prevalence 1/200–1/250), but the condition is underdiagnosed [41]. FH is caused by monogenetic mutations in the *LDLR*, *APOB*, or *PCSK9* genes and carries a tenfold increased risk of coronary artery disease. These patients will often present with early onset ASCVD, as the condition itself is usually asymptomatic. The FH score of the Dutch Lipid Clinic Network (www.fhscore.eu) can establish a diagnosis of FH and categorize the probability of FH. Importantly, a simple LDL-C concentration-based approach for the identification of individuals with FH may provide similar diagnostic accuracy as the FH score [42, 43]. In children, these scores have a poor sensitivity, such that genetic testing is recommended if FH is suspected [44]. Early identification of FH patients and intensive lipid management is crucial to reduce the cardiovascular risk.

### Individuals < 40 years

The SCORE2 algorithm is not validated in this group. In these patients, *lifetime* cholesterol burden and associated cardiovascular risk need to be considered. For patients as young as 35 years free from cardiovascular disease, lifetime cardiovascular risk may be calculated using the LIFE-CVD2 model (www.u-prevent.com). This tool also allows the user to evaluate the effects of various pharmacological and non-pharmacological interventions on lifetime cardiovascular risk [45].

The risk associated with ApoB-containing lipoproteins is influenced by both the concentration of these lipoproteins and the cumulative duration of exposure (i.e., cholesterol burden). Maintaining optimal lipid levels throughout life aims to keep circulating levels of LDL and other ApoB–containing lipoproteins low, thereby reducing the number of particles retained in the arterial wall and slowing the development of atherosclerotic plaques. Since ApoB–containing lipoproteins exert both causal and time-dependent effects on ASCVD risk, the most effective preventive approach is to achieve and sustain healthy lipid levels from an early age. Cholesterol burden may be illustrated in terms of “cholesterol mg-years” (= approximate LDL-C level in mg/dL × years at this level) [11]. After approx. 5000 cholesterol mg-years, the risk of MI reaches 1% and then continues to rise log-linearly if cholesterol levels remain the same. In a patient with 125 mg/dL LDL-C levels, this risk level is attained at approximately 40 years of age. However, particularly for younger adults, it should be pointed out that lowering LDL-C can significantly reduce the risk of ASCVD and plaque burden given the longer time frame. For instance, in a large meta-analysis, lowering LDL-C by 1 mmol/l was associated with a stronger reduction in mortality from ischemic heart disease in people 40–49 (hazard ratio 0.44 [95% CI 0.42–0.48]) compared to those 50–69 years (0.66 [CI 0.65–0.68]) and 70–89 years (0.83 [CI 0.81–0.85]) [46]. These concepts question the idea of “normal” LDL-C levels, since, given enough time, even normal levels can lead to the manifestation of ASCVD. “Normal” denotes statistical commonality within the general population and should not be conflated with “healthy”. In fact, there are no safety matters to date of lowering LDL-C to very low levels [47], as, for instance, individuals with *PCSK9* loss-of-function mutations maintain LDL-C levels as low as 14 mg/dL and are healthy [48]. Furthermore, in patients living outside Western societies, normal LDL-C levels are between 50–70 mg/dL [49].

Moreover, patients may be up-classified in the context of important *risk modifiers*, which are not reflected in the current SCORE2 classification. These risk modifiers are detailed in the 2025 dyslipidaemia guideline update [39]. Risk modifiers are particularly relevant in moderate- and low-risk situations. If risk modifiers are present, the threshold to initiate pharmacological lipid-lowering therapy decreases. Important risk modifiers include psychosocial stress, ethnicity, imaging (e.g., coronary artery calcium scanning and carotid ultrasound), family history, obesity and socioeconomic determinants, pre-eclampsia and other hypertensive pregnancy disorders, HIV infection, chronic inflammatory disorders and biomarkers such as elevated high-sensitivity C-reactive protein (hsCRP) and elevated Lp(a) [36, 39]. In the following paragraphs, we would like to highlight the risk modifiers Lp(a), chronic inflammatory conditions, and hypertriglyceridaemia due to their prominent roles.

### Lipoprotein(a)

The risk of ASCVD increases continuously with rising Lp(a) levels. For instance, Lp(a) levels above 100 mg/dL approximately double cardiovascular risk independently of baseline risk [28]. For practical purposes, however, the 2022 ESC/EAS consensus document recommends a rule-in and rule-out approach: Lp(a) < 30 mg/dL (< 75 nmol/L) should be “ruled out” as a significant risk modifier. Lp(a) > 50 mg/dL (> 125 nmol/L) should be “ruled in”. Lp(a) levels of 30–50 mg/dL (75–125 nmol/L) constitute a “grey zone” and should be interpreted in the context of other cardiovascular risk factors and overall risk [28]. The recent 2025 update of the 2019 guidelines for the management of dyslipidaemias also recommends consideration of Lp(a) concentrations above 50 mg/dL as a risk-enhancing factor [39]. In the primary prevention setting, it may be helpful to use a risk calculator (http://www.lpaclinicalguidance.com) which confers how much individual Lp(a) levels increase the risk of ASCVD. With increasing Lp(a) concentrations, more aggressive risk factor management is recommended. In the absence of specific Lp(a)-lowering therapies, increased efforts should be made to achieve LDL-C, blood pressure, and glucose targets. PCSK9 inhibitors lower Lp(a) by approximately 25% [50]. However, this effect is felt to be too small to efficiently lower event rates in patients with elevated Lp(a) (see below). Moreover, PCSK9 inhibitors are not registered, and current guidelines do not recommend using PCSK9 inhibitors for this purpose [28, 39]. In patients with elevated Lp(a), cascade testing is recommended, particularly in cases of family history of ASCVD. This should be limited to Lp(a) measurement as there is no role for genetic testing for Lp(a) [28].

### Chronic inflammatory conditions

Chronic inflammatory conditions are significant risk modifiers in ASCVD. These include rheumatoid arthritis (RA), systemic lupus erythematosus (SLE), psoriasis, and inflammatory bowel disease (IBD). Patients with RA and SLE have a markedly elevated risk of ASCVD, comparable to that seen in diabetes mellitus. In a broader sense, patients with HIV should also be grouped into this category as similar underlying mechanisms apply [51]. This increased risk is attributed to both traditional cardiovascular risk factors and the chronic systemic inflammation inherent in these conditions. Inflammatory pathways, including those involving cytokines such as TNF-α, IL-1β, and IL-6, play a crucial role in the pathogenesis of both chronic inflammatory diseases and atherosclerosis [22, 52, 53]. Regular cardiovascular risk assessment and aggressive management of traditional risk factors in patients with chronic inflammatory diseases are recommended. Regarding patients with HIV, a recent study demonstrated a significant risk reduction of major adverse cardiac events in low-moderate risk patients treated with pitavastatin, strengthening the argument for a liberal prescription of statins to lower LDL-cholesterol in this subgroup [54]. Accordingly, the recent ESC dyslipidaemia guideline update recommends statin therapy in primary prevention for HIV patients ≥ 40 years irrespective of estimated cardiovascular risk and LDL-C levels [39]. Treating the underlying disease using anti-inflammatory therapies may also reduce cardiovascular risk in inflammatory conditions. For instance, TNF inhibitors have shown potential in reducing cardiovascular events by controlling systemic inflammation [36, 55, 56].

### Hypertriglyceridaemia (HTG)

Hypertriglyceridaemia (HTG) should also be considered a risk modifier, particularly in patients with otherwise optimized LDL-C, where it may confer residual risk. If HTG is diagnosed, patients should first be evaluated for secondary causes including but not limited to alcohol abuse, pregnancy, diet (high glycaemic load), obesity, type 2 diabetes, hypothyroidism, renal disease, and medications [57, 58]. Severely elevated TG levels > 500 mg/dL and particularly > 1000 mg/dL increase the risk of pancreatitis. If a secondary cause has been excluded, these patients usually have a polygenic disposition or—in rare cases—a monogenic cause (familial chylomicronaemia syndrome) [24, 59]. Apart from treating secondary causes of HTG, we suggest that lifestyle modifications (limiting alcohol consumption, weight loss), dietary changes (low in refined carbohydrates and fructose), and increased physical activity should be the first-line approach to managing HTG [29]. In high-risk and very-high-risk patients, statin therapy is indicated to reduce global cardiovascular risk. Furthermore, high-dose icosapent ethyl (2 × 2 g/d) is an option, although currently not available in Germany [60]. Volanesorsen may be an option for patients with severe HTG (> 750 mg/dL) and genetically proven familial chylomicronaemia syndrome, the main goal being, however, to reduce the risk of pancreatitis [39]. These patients require close surveillance because of thrombocytopenia as an adverse effect, among others [61].

## Lifestyle modification

Lifestyle management is the cornerstone of the treatment and prevention of dyslipidemia, forming the foundation upon which pharmacologic therapy is built. Lifestyle modification can significantly improve lipid profiles and reduce cardiovascular risk. Important strategies include adopting a healthy dietary pattern—such as the Mediterranean or DASH diet—relying on fruits, vegetables, whole grains, legumes, nuts, and sources of unsaturated fats while limiting saturated and trans fats, refined carbohydrates, and added sugars [62, 63]. Regular physical activity, consisting of at least 150 min of moderate-intensity or 75 min of vigorous-intensity aerobic exercise per week, enhances HDL cholesterol, lowers triglycerides, and improves insulin sensitivity. Weight reduction in overweight or obese individuals has a dose-dependent effect on lowering LDL cholesterol and triglycerides. Smoking cessation and moderation of alcohol intake further contribute to lipid optimization and vascular health [29, 36].

## Lipid-lowering therapy

**Fig. 1:**
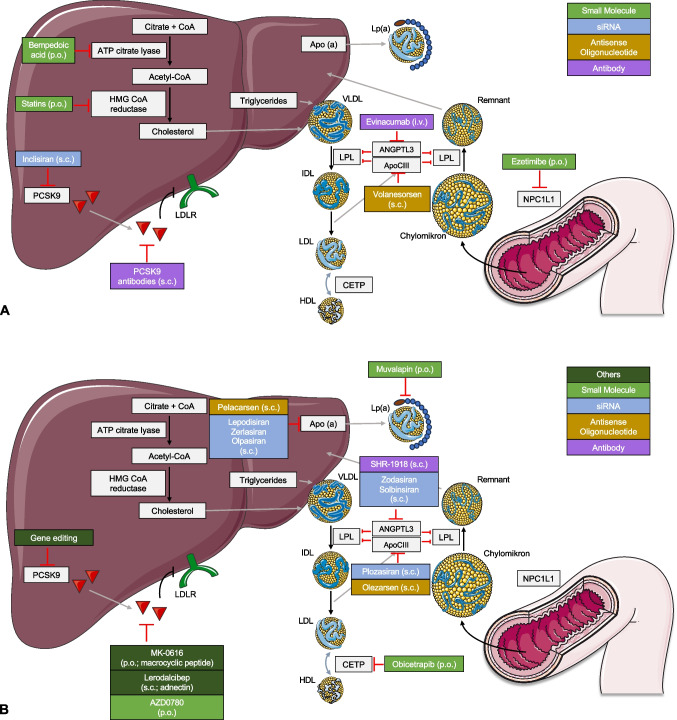
Schematic overview of lipid-lowering drugs currently available (**A**) or in advanced clinical trials (**B**) and their mechanisms of action**.** Apo(a)—apolipoprotein (a), ApoCIII—apolipoprotein C-III, ANGPTL3—angiopoietin-like protein 3, ATP—adenosine triphosphate, CETP—cholesteryl ester transfer protein, CoA—coenzyme A, HMG—3-hydroxy-3-methylglutaryl, HDL—high-density lipoprotein, IDL—intermediate-density lipoprotein, LDL—low-density lipoprotein, LDLR—LDL-receptor, Lp(a)—lipoprotein (a), LPL—lipoprotein lipase, NPC1L1—Niemann-Pick C1-like 1, PCSK9—proprotein convertase subtilisin/kexin type 9, and VLDL—very-low-density lipoprotein. Images and icons shown were provided by Servier Medical Art (https://smart.servier.com/), licensed under CC BY 4.0 (https://creativecommons.org/licenses/by/4.0/)

Once the decision to initiate pharmacological lipid-lowering therapy has been made depending on underlying risk, there is an arsenal of drugs available. Figure 1 provides an overview of lipid-lowering drugs discussed below currently available (panel A) or in advanced clinical trials (panel B) and their mechanisms of action. In addition, the recent guideline update provides a valuable figure detailing the expected LDL-C lowering effect of different therapeutic agents alone and in combination [39].

Many patients use dietary supplements or vitamins to reduce their cardiovascular risk; however, profound effects on LDL-C concentrations and outcome data are missing [64, 65]. The 2025 updated dyslipidaemia guidelines therefore advise against treatment with dietary supplements or vitamins to reduce ASCVD risk (class III recommendation) [39].

The authors support a pharmaceutical approach to all patients in the “high risk” or “very-high risk” categories who do not meet their respective LDL-C targets, in line with international guidelines [29, 66]. The cornerstone of current lipid-lowering therapies is statins. Highly potent statins (rosuvastatin, atorvastatin) should be favoured over lower potency statins (e.g., simvastatin and fluvastatin). Statins reduce cardiovascular events and are usually well tolerated, with reductions of LDL-C of ~ 50% with high-intensity statin therapy [29]. For every 40 mg/dL LDL-C lowering, statins reduce the relative risk of major adverse cardiac events by approximately 25% in patients with and without manifestations of ASCVD [67–69].

Physicians should be aware of potential adverse effects and limitations when prescribing statins. Statin intolerance primarily manifests as statin-associated muscle symptoms (SAMS), which include myalgia, myopathy, and, in extremely rare cases, rhabdomyolysis. Myalgia is the most common presentation, characterized by muscle pain, tenderness, or weakness without significant elevation in creatine kinase (CK) levels. The incidence of statin intolerance varies significantly between major clinical trials and real-life settings. In randomized controlled trials, the prevalence of statin intolerance is reported to be around 4.9%. In contrast, observational cohort studies and real-world data suggest a much higher prevalence, approximately 17% [70]. Several factors contribute to this discrepancy. Clinical trials often have strict inclusion and exclusion criteria, leading to the selection of healthier and more compliant patients. In real-life settings, patients are more diverse, with a higher prevalence of comorbidities and polypharmacy, which can increase the risk of adverse effects [70, 71]. Moreover, the “nocebo” effect, which reflects the patients’ expectation of adverse effects yielding the perception of symptoms, is more pronounced in real-world practice. This effect is less likely to be observed in blinded RCTs [72].

Additionally, drug interactions may be of concern, e.g., in HIV or patients after organ transplant receiving immunosuppression. In these cases, pravastatin and fluvastatin are (less potent) alternatives due to minimal metabolism by cytochrome P450 enzymes [73].

If target LDL-C levels are not attained by statin monotherapy, we advocate the addition of ezetimibe, as its mechanism of action is complementary to statins via inhibition of Niemann-Pick C1-Like 1 (NPC1L1) protein, which plays a crucial role in cholesterol absorption in the intestine. The combination of ezetimibe with a high-intensity statin can reduce LDL-C by approximately 65% [29].

Another novel orally available LDL-C-lowering drug is bempedoic acid, which lowers LDL-C by approximately 20–25% by inhibiting the ATP citrate lyase [74] and is now, after the positive CLEAR outcomes trial has demonstrated cardiovascular benefit, also recommended by the recently updated ESC guidelines for the management of dyslipidaemias for patients with statin intolerance or those not at LDL-C target [39, 74].

If the LDL-C targets are not achieved with oral combination therapy, the PCSK9 antibodies evolocumab or alirocumab, and the small-interfering RNA inclisiran can be prescribed with an expected LDL-C lowering of ~ 50–60% [29]. Widespread implementation of the use of PCSK9 inhibitors is limited by comparably high treatment costs and restrictions for prescription. For inclisiran, two major cardiovascular outcomes trials are ongoing. For evolocumab and alirocumab, large outcomes trials have shown a 15% relative reduction in risk for both drugs. Only recently, a second large outcomes trial for evolocumab has been published. In the VESALIUS-CV trial, patients without previous myocardial infarction or stroke (but with atherosclerosis or diabetes) were treated with evolocumab or placebo. To meet inclusion criteria, patients had to have an LDL-C > 90 mg/dL while on optimized standard lipid-lowering therapy. Patients treated with evolocumab had an LDL-C reduction of 55%, which led to significant reductions in the 3-point (25%) and the extended 4-point (19%) MACE endpoints (hazard ratios, 0.75; 95% CI 0.65 to 0.86; *p* < 0.001 and 0.81; 95% CI, 0.73 to 0.89; *p* < 0.001). There was no heterogeneity in subgroups and no group differences in adverse events over a median of 4.6 years of follow-up. This study indicates that patients at elevated cardiovascular risk, but without a previous cardiovascular event, derive the same benefit from treatment with evolocumab as those with previous MI or stroke [75].

Instead of the historically recommended step-wise approach, initial combination therapy (e.g., statin + ezetimibe) should be used for patients with recent acute coronary syndromes (ACS), particularly if statin monotherapy is not expected to achieve LDL-C targets. This is in line with a registry analysis from Allahyari et al. demonstrating that a majority of patients would not attain current treatment targets on statin therapy alone [76]. Patients with an ACS already receiving lipid-lowering therapy should undergo treatment intensification during the index hospitalisation. These recommendations are key novel recommendations of the recent 2025 update of the 2019 dyslipidaemia guidelines. The rationale is founded on data demonstrating improved outcomes in patients who achieve their LDL-C targets early, for instance from the SWEDEHEART registry. These concepts can be summarized as “strike early and strong” [76] and “the sooner, the lower, the better” [39, 77, 78]. Notably, this approach in the post-ACS setting was proven successful in studies by Makhmudova et al. and by Hagiwara et al. before the recent ESC/EAS guidelines update [79, 80].

Additionally, there are drugs specifically indicated for the very rare condition of homozygous FH. These include the angiopoietin-like protein 3 (ANGPTL3) inhibitor evinacumab and the microsomal triglyceride transfer protein (MTP) inhibitor lomitapide. However, lomitapide is not available in Germany, and there is concern of hepatotoxicity [81]. According to the German federal joint committee (G-BA), both drugs received the label “additional benefit not proven”. This usually precludes funding under statutory health insurance. This restriction is in conflict with the class IIa recommendation provided for evinacumab in the recent ESC guideline update for the management of dyslipidaemias [39].

Moreover, there are ongoing research efforts in the field of lipid-lowering drugs. For instance, obicetrapib is a novel cholesteryl ester transfer protein (CETP) inhibitor. It reduces LDL-C, ApoB, and Lp(a) while increasing HDL-C. Obicetrapib is currently being evaluated in phase III trials for its efficacy and safety in patients with ASCVD or heterozygous FH whose LDL-C levels remain high despite maximally tolerated lipid-modifying therapies [82].

Gene editing, particularly using the CRISPR/Cas9 system, offers a permanent solution by directly modifying the DNA sequence of target genes. This approach has shown promise in preclinical studies and early-phase clinical trials, particularly for rare conditions, e.g., homozygous FH, but may also benefit patients with more common conditions such as heterozygous FH. Target genes being investigated include PCSK9, ANGPTL3, LDLR, and APOC3 [83]. For example, preclinical studies have demonstrated that CRISPR/Cas9-mediated editing of the PCSK9 gene in the liver can lead to a near-complete knockdown of PCSK9 protein, resulting in significant reductions in LDL-C levels. In nonhuman primates, a single infusion of lipid nanoparticles carrying CRISPR base editors targeting PCSK9 resulted in a ~ 90% reduction in blood PCSK9 levels and about 60% reduction in LDL-C levels with effects lasting for at least 8 months [84].

Although specific Lp(a) lowering therapies are not yet available, there are strong ongoing research efforts and results of the first ongoing Phase III trials are expected in 2026 (Table 1). The extent of Lp(a) lowering needed to achieve a clinically meaningful benefit remains uncertain: Mendelian randomization studies have suggested that Lp(a) lowering of 66 to 100 mg/dl may be required to achieve meaningful improvements in cardiovascular outcomes [85, 86]. In the meantime, clinicians should strive to manage all other risk factors in patients with Lp(a), particularly those with established ASCVD. Clinicians should be aware that while statins and PCSK9 inhibitors increase LDLR expression, statins may increase Lp(a) levels slightly, without expected negative impact on cardiovascular risk, whereas PCSK9 inhibitors decrease Lp(a) concentration by 15–30% [87, 88].

**Table 1:** Lp(a)-lowering drugs in advanced development stages

	**Mechanism**	**Lp(a) reduction in phase II trials**	**Phase III trials (ongoing)**
Lepodisiran	Hepatocyte directed siRNA	Up to 94% [89]	ACCLAIM-Lp(a)
Olpasiran	Hepatocyte directed siRNA	Up to 101% [90]	OCEAN (a)
Pelacarsen	Hepatocyte-directed antisense oligonucleotide	Up to 80% [91]	HORIZON
Zerlasiran	Hepatocyte directed siRNA	Up to 96% [92]	Not commenced
Muvalaplin	Oral inhibitor of Apo(a) and ApoB interaction	Up to 86% [93]	MOVE-Lp(a)

Lipoprotein apheresis (LA) can be a therapy of last resort for patients with refractory hyperlipidaemia (LDL-C and Lp(a)). In most cases, it requires established ASCVD, except for patients with homozygous FH. Although observational studies indicate benefit, there are no large randomized controlled trials to support the efficacy of LA, and the procedure is costly and stressful for patients, requiring regular venous access, partly by a shunt. Due to the availability of PCSK9 inhibitors, the number of patients on LA for LDL-C lowering has decreased significantly. LA is, however, frequently required for patients with homozygous FH, who cannot be managed sufficiently with medical therapy. In Germany, LA for Lp(a) lowering requires an Lp(a) level > 60 mg/dL and progressive ASCVD despite optimal control of all other cardiovascular risk factors, including LDL-C [94, 95].

Novel Lp(a) lowering drugs such as Pelacarsen may avoid LA, which was investigated in a recently completed multicentre trial (NCT05305664).

## Conclusion

Hyperlipidaemia is a prominent cardiovascular risk factor worldwide. Efforts should be made to identify patients at risk at an early age to prevent or delay the occurrence of ASCVD via targeted interventions. Once ASCVD has been diagnosed, patients require rapid lipid-lowering and strict control of LDL-C and other risk factors. The basis of lipid-lowering is a lifestyle intervention. Statins are the initial drugs of choice. Early combination therapy with ezetimibe, bempedoic acid, and PCSK9 inhibitors is recommended for high-risk patients and for those who are far from achieving their LDL-C targets. Combination therapy allows > 90% of patients to achieve their LDL-C target.

Ongoing research efforts may provide the opportunity to specifically address lipoproteins contributing to residual cardiovascular risk such as elevated triglycerides and Lp(a) and are likely to improve the individualized treatment of patients at risk.
